# Low Serum Phosphorus Correlates with Cerebral Aβ Deposition in Cognitively Impaired Subjects: Results from the KBASE Study

**DOI:** 10.3389/fnagi.2017.00362

**Published:** 2017-11-06

**Authors:** Jong-Chan Park, Sun-Ho Han, Min S. Byun, Dahyun Yi, Jun Ho Lee, Kyua Park, Dong Young Lee, Inhee Mook-Jung

**Affiliations:** ^1^Department of Biochemistry and Biomedical Sciences, College of Medicine, Seoul National University, Seoul, South Korea; ^2^Neuroscience Research Institute, College of Medicine, Seoul National University, Seoul, South Korea; ^3^Institute of Human Behavioral Medicine, Medical Research Center Seoul National University, Seoul, South Korea; ^4^Department of Neuropsychiatry, Seoul National University Hospital, Seoul, South Korea; ^5^Department of Biology, College of Arts & Sciences, University of Pennsylvania, Pennsylvania, PA, United States; ^6^Department of Psychiatry, Seoul National University College of Medicine, Seoul, South Korea

**Keywords:** phosphorus, Alzheimer's disease, mild cognitive impairment, β-amyloid, PiB-PET, KBASE, blood-based biomarker

## Abstract

Alzheimer's disease (AD), characterized by progressive cognitive decline, is the most prevalent neurodegenerative disease in the elderly. Cerebral β-amyloid (Aβ) deposition is the major pathological hallmark of AD. Recent studies also have shown that the serum level of phosphorus correlates to the risk of incident dementia. To date, the linkage between cerebral Aβ deposition and the serum phosphorus level remains unknown. In this study, we analyzed the levels of serum phosphorus in 109 mild cognitive impairment (MCI) and 73 AD dementia (ADD) subjects. All subjects underwent Pittsburgh compound B positron emission tomography (PiB-PET) imaging to measure cerebral Aβ deposition. The results with Aβ deposition was compared with the serum levels of phosphorus. The subjects with cerebral Aβ deposition showed lower levels of serum phosphorus than those without Aβ deposition. Furthermore, multiple regression analyses showed that a low level of serum phosphorus correlated with cerebral Aβ deposition, even when age, sex, apolipoprotein E ε4 genotype, and MMSE z-score were controlled for. Serum levels of other ions, including calcium, iron, zinc, and copper, showed no such correlation. In conclusion, our results suggest that the serum level of phosphorus may be used as an easily accessible blood biomarker for cerebral Aβ deposition in a cognitively impaired population.

## Introduction

Alzheimer's disease (AD) is one of the most disastrous neurodegenerative diseases and the most prevalent cause of dementia in elder population. Since methods for early diagnosis and for treatment of AD are insufficient, diverse problems emerge as the aged population increases in modern societies. Cerebral β-amyloid (Aβ) plaques and neurofibrillary tangles (NFT) are characteristics of AD (Hardy and Higgins, 1992; Scheuner et al., 1996; Wisniewski et al., 1997; Murphy and LeVine, 2010; Bloom, 2014). Particularly, the abnormal production and impaired clearance of Aβ contribute to Aβ accumulation, which is aggregated into plaques (Mawuenyega et al., 2010; Querfurth and LaFerla, 2010). The cerebral accumulation of Aβ is closely associated with cellular toxicity, synaptic dysfunction, aberrant neuronal activity, and destabilization of neural networks (Palop and Mucke, 2010). Even though Aβ pathology may not necessarily correlate with cognitive decline in a linear manner, accumulating evidence indicates that cerebral Aβ plays a critical role in AD pathogenesis and that neuritic plaques are specific characteristics of AD (Hardy and Higgins, 1992; Wisniewski et al., 1997; Nelson et al., 2009). Consequently, detection of Aβ plaques in brains is important not only for early diagnosis of AD but also for the differential diagnosis of AD from other causes of cognitive impairment. As such, it is crucial to determine whether cognitively impaired subjects, including mild cognitive impairment (MCI) or dementia patients, have cerebral Aβ deposition in order to diagnose the underlying AD accurately. Although Aβ deposition can be measured by positron emission tomography (PET) using radioactive ligands, like Pittsburgh compound B (PiB), it is very expensive and not commonly accessible (Kang et al., 2016; Park et al., 2017). Furthermore, although several cerebrospinal fluid (CSF) biomarkers, including CSF Aβ, show correlations with cerebral Aβ, sampling human CSF is an invasive procedure, and the inter-institutional reliability of CSF AD biomarker measurement is suboptimal. Therefore, discovery of easily accessible biomarkers that reflect the degree of cerebral Aβ deposition would be very beneficial.

Since some ions, including phosphorus, magnesium, and calcium, are highly involved in the pathogenesis of age-related neurological abnormality, many researchers have been interested in the relationship between these ions and neurodegeneration (Glick, 1990; Durlach et al., 1997; Andrási et al., 2005; Canzoniero and Snider, 2005; Mattson, 2007). Compared with control brains, various regions of AD brains were shown to contain increased levels of aluminum but decreased levels of magnesium as well as phosphorus (Glick, 1990; Andrási et al., 2005). In addition, AD patients had relatively lower levels of plasma magnesium, copper, zinc, iron, and selenium (Vural et al., 2010). Among these substances, phosphorus is abundant in human brains (Andrasi et al., 1995) and is an integral element essential for controlling numerous physiologic processes of the human body. Studies have shown that various processes such as energy storage, bone and muscle production, hormonal balance, and brain metabolism require appropriate level of phosphorous (Fiske and Subbarow, 1929; Bourgoignie, 1982; Shi et al., 2003; Takeda et al., 2012; Li et al., 2017). In addition, AD pathogenesis involves phosphorus in many key processes such as hyperphosphorylation of tau and accumulation of Aβ (Yumoto et al., 2009; Bloom, 2014). A recent study also showed that phosphorus dendrimer interacts with amyloid peptide and microtubule associated protein (MAP) tau in such a way that it affects the aggregation process of Aβ and tau (Wasiak et al., 2012). Furthermore, the level of phosphorus was significantly reduced in various regions of AD brains, including cortex entorhinalis and cortex frontalis basalis (Andrási et al., 2005). However, the relationship between peripheral level of phosphorus and AD pathogenesis remains unclear.

In this study, we investigated whether serum level of phosphorus is associated with cerebral deposition of Aβ in cognitively impaired subjects. We measured cerebral Aβ deposition in 182 cognitively impaired subjects using PiB-PET and compared the results with their serum levels of phosphorus. We show that the deposition of cerebral Aβ correlates with the decreased serum levels of phosphorus.

## Materials and methods

This study was part of the Korean Brain Aging Study for the Early Diagnosis and Prediction of Alzheimer's Disease (KBASE), an ongoing prospective cohort study which started its recruitment in 2014 and aimed to find biomarkers for AD as well as to determine risk factors for AD-related functional and structural brain changes. This work was approved by the Institutional Review Board (IRB) of the Seoul National University Hospital and SMG-SNU Boramae Medical Center, South Korea. All subjects or their legal representatives provided their written informed consent after being fully informed about the study.

### Participants

As of August 2016, a total of 182 subjects who were 55 years or older participated in the study. Among the subjects, 109 had mild cognitive impairment (MCI), and 73 had clinically diagnosed AD dementia (ADD). They underwent comprehensive KBASE baseline assessment including clinical examination, neuropsychological assessments, neuroimaging, including magnetic resonance imaging (MRI), PiB-PET, and blood tests. Individuals with MCI (age 55–90 [inclusive]) met the following criteria: (a) memory complaint reported by themselves or reported by an informant or a clinician; (b) presence of objective memory impairment; (c) intact functional activities; and (d) non-demented. All MCI subjects had a global CDR score of 0.5. With respect to criterion (b), MCI individuals scored at least 1.0 standard deviation (SD) below the respective age, education, and gender-specific mean on at least one of the four memory tests that were part of the Consortium to Establish a Registry for Alzheimer's Disease (CERAD) neuropsychological battery (namely, Word List Memory, Word List Recall, Word List Recognition, and Constructional Recall test) (Lee et al., 2004). Inclusion criteria for patients with ADD (age 55–90 [inclusive]) were: (a) meet both criteria of the Diagnostic and Statistical Manual, 4th Edition for dementia (Americal Psychiatric Association, 2000) and the criteria for probable ADD set according to the National Institute of Aging and Alzheimer's Association (NIA-AA) guidelines (McKhann et al., 2011), and (b) have a global CDR score of 0.5 or 1. The exclusion criteria were: (a) presence of any psychiatric or neurological disorders that could affect mental function, (b) severe communication problems that would create difficulty in a assessment or brain scan, (c) contraindications for MRI scanning, (d) absence of a reliable informant, or (e) illiteracy.

### Clinical and neuropsychological assessment

All participants underwent standardized clinical assessment by trained psychiatrists, based on the KBASE protocol, which corresponded with the Korean version of the Consortium to Establish a Registry for Alzheimer's Disease Assessment Packet (CERAD-K) (Lee et al., 2002). In addition, participants underwent KBASE neuropsychological assessment by trained neuropsychologists while incorporating the CERAD neuropsychological battery (Lee et al., 2004), which contained the Mini-Mental State Examination (MMSE). Due to the influence of gender, age, and education on MMSE score (Piccinin et al., 2013), we used the z-score of MMSE instead (Lee et al., 2004). Body mass index (BMI) was calculated for all participants from their heights and weights. Through a weekly clinical review panel meeting (chaired by DL) with a clinical review panel composed of several geriatric psychiatrists, clinical neuropsychologists, and psychometrists, individuals were allocated to either MCI group or ADD group. Ineligible individuals were excluded.

### PIB-PET

Participants underwent three-dimensional PiB-PET imaging and T1-weighted MR using a Biograph mMR scanner (Siemens, Washington DC, USA). Forty minutes after intravenous injection of 555 MBq of ^11^C-PiB, a 30-min emission scan was obtained. The data were reconstructed into a 256 × 256 image matrix using iterative methods (6 iterations with 21 subsets) and were corrected for uniformity, ultrashort echo time (UTE)-based attenuation, and decay reduction. Sagittal T1-weighted images (repetition time = 1,670 ms; echo time = 1.89 ms; field of view, 250 mm; 256 × 256 matrix with 1.0 mm slice thickness) were acquired. Images were pre-processed using Statistical Parametric Mapping 8 (SPM8) implemented in Smith and Barton (2014) (MathWorks, Natick, MA). PiB-PET data were co-registered to the individual T1 images and transformation parameters to a standard Montreal Neurological Institute (MNI) template were computed. Individual Brain Atlases using Statistical Parametric Mapping Software (IBASPM) software was used for the inverse transformation parameters, which transformed the coordinates from the automatic anatomic labeling (AAL) 116 atlas (Tzourio-Mazoyer et al., 2002) into an individual space for each subject (resampling voxel size = 1 × 0.98 × 0.98 mm). Only the gray matter (GM) was used for extraction by applying GM mask to each individual in order to exclude white matter and CSF space.

Based on previous studies (Jack et al., 2008; Reiman et al., 2009; Choe et al., 2014; Park et al., 2017), the cerebral regional mean ^11^C-PiB uptake values were calculated using the individual AAL116 atlas from the T1-coregistered PiB-PET images and the cerebellar gray matter ^11^C-PiB uptake values were used for the quantitative normalization. To determine the regions of interest (ROIs), the AAL algorithm and a region-combining method (Reiman et al., 2009) were applied and brain regions were divided into the frontal, lateral parietal, posterior cingulate-precuneus (PC-PRC), and lateral temporal regions, where prominent ^11^C-PiB retention has been reported (Klunk et al., 2004). The standardized uptake value ratio (SUVR) of each ROI was obtained by dividing the mean value for all voxels within the ROI by the mean cerebellar gray matter uptake value. Subjects were classified as PiB positive (PiB+) if the SUVR value exceeded 1.4 in at least one of the four ROIs (i.e., frontal, lateral temporal, lateral parietal, and PC-PRC) or as PiB negative (PiB-) if the SUVR values of all four ROIs were equal to or less than 1.4 (Reiman et al., 2009; Choe et al., 2014). Being classified as PiB− would mean that subjects have negative amyloid deposition, while being classified as PiB+ would mean that subjects have positive amyloid deposition.

### Blood sampling and quantification of serum phosphorus, calcium, iron, zinc, and copper

Blood samples were taken via venipuncture in the morning (around 9 am) after overnight fasting and were collected into serum separator tubes (Becton, Dickinson and Co., Franklin Lakes, NJ, USA). The tubes were stored at room temperature (RT) for 30 min and then were centrifuged at 1,300 g for 10 min at RT. Separated serum was used for assays. Serum phosphorus, calcium, and iron levels were measured at Seoul Clinical Laboratories, using an enzymatic colorimetric method (ADVIA 1800 Autoanalyzer; Siemens, Washington DC, USA). The levels of zinc and copper were determined by inductively coupled plasma-mass spectroscopy (ICP-MS) using a Varian 820-MS ICP mass spectrometer (Bruker, Victoria, Australia). Additionally, apolipoprotein E (APOE) genotyping was done by using the genomic DNA, as previously described (Wenham et al., 1991).

### Statistics

To investigate the relationship between serum phosphorus and global cerebral Aβ deposition, partial correlation analysis was used, with correction for the influence by the different covariates (CV; age and sex). Variables were logarithmically transformed to adjust skewness of the distribution and to normalize variance. To compare the serum phosphorus level between PiB− and PiB+ with statistical control for the effects of CV (age and sex), analysis of covariance (ANCOVA) was performed, and *p*-values were obtained by pairwise comparison. For the independent association between serum phosphorus level and global cerebral Aβ deposition, multiple regression analyses were conducted after controlling for age, sex, ApoE ε4 carrier status, calcium, MMSE z-score, and others. To identify the relationship between serum phosphorus level and risk of accumulation of global cerebral Aβ, relative risk (95% confidence interval) tests were conducted. In addition, we carried out logistic regression analysis followed by receiver operating characteristic (ROC) curve analysis to show the discrimination power of ApoE ε4 carrier status and phosphorus on PiB positivity. The predicted probabilities were used to calculate the discrimination power for the prediction of PiB positivity. Comparison of ROC curves was performed according to a previous report, DeLong et al. (1988). For the demographic data of subjects (Table 1), independent *t*-tests were performed to compare values (age, education, MMSE z-score, and global amyloid deposition). Chi-squared tests were carried out to evaluate the intergroup differences of categorical variables (sex, CDR score, and ApoE ε4 carrier status). All statistical analyses were performed using Medcalc 17.2 (Medcalc Software, Ostend, Belgium). P values were stated in each figure or table to indicate statistical significance as appropriate.

**Table 1:** Demographic and clinical characteristics of participants.

	**MCI (*n* = 109)**	**ADD (*n* = 73)**	***P*-value**
Gender, M/F	36/73	23/50	0.8304^a^
Age, years	73.98 ± 0.6	72.73 ± 0.9	0.2559^b^
Education, years	9.70 ± 0.4	9.37 ± 0.6	0.6628^b^
MMSE z-score	−1.22 ± 0.1	−3.13 ± 0.2	<0.0001^b^^*^
Global CDR, *N* (%)			<0.0001^a^^*^
0.5	109 (100%)	24 (32%)	
1	0 (0%)	49 (68%)	
ApoE ε4 carrier, *N* (%)	35 (32%)	41 (56%)	0.0013^a^^*^
Global Aβ deposition (SUVR)	1.53 ± 0.1	1.87 ± 0.1	<0.0001^b^^*^
PiB positivity, %	50.5%	78.1%	0.0002^a^^*^

*Data were presented as Mean ± SEM or N (%)*.

*MCI, mild cognitive impairment; ADD, Alzheimer's disease dementia; PiB, Pittsburgh compound B; ±, PiB positivity; SEM, standard error of mean; n, number of subjects; MMSE, mini-mental state examination; MMSE z score, a revised value of the MMSE score with consideration for age, gender, and education level; CDR, clinical dementia rating; ApoE, Apolipoprotein E; SUVR, standardized uptake value ratio*.

a P, significance by chi-squared test;

b P, significance by independent t-test;

* *p < 0.05*.

## Results

### Demographic data of subjects

A total of 182 subjects (109 MCI subjects and 73 ADD subjects) were included in this study. Table 1 shows the demographic and clinical characteristics of the participants (Table 1). Amongst the MCI subjects, 50.5% were PiB+ (*n* = 55), whereas 78.1% of ADD patients were PiB+ (*n* = 57). No differences were observed in age (*p* = 0.256) and sex (*p* = 0.830) between MCI and ADD groups (Table 1).

### Relationship between the serum level of phosphorus and the global deposition of cerebral amyloid

**Figure 1:**
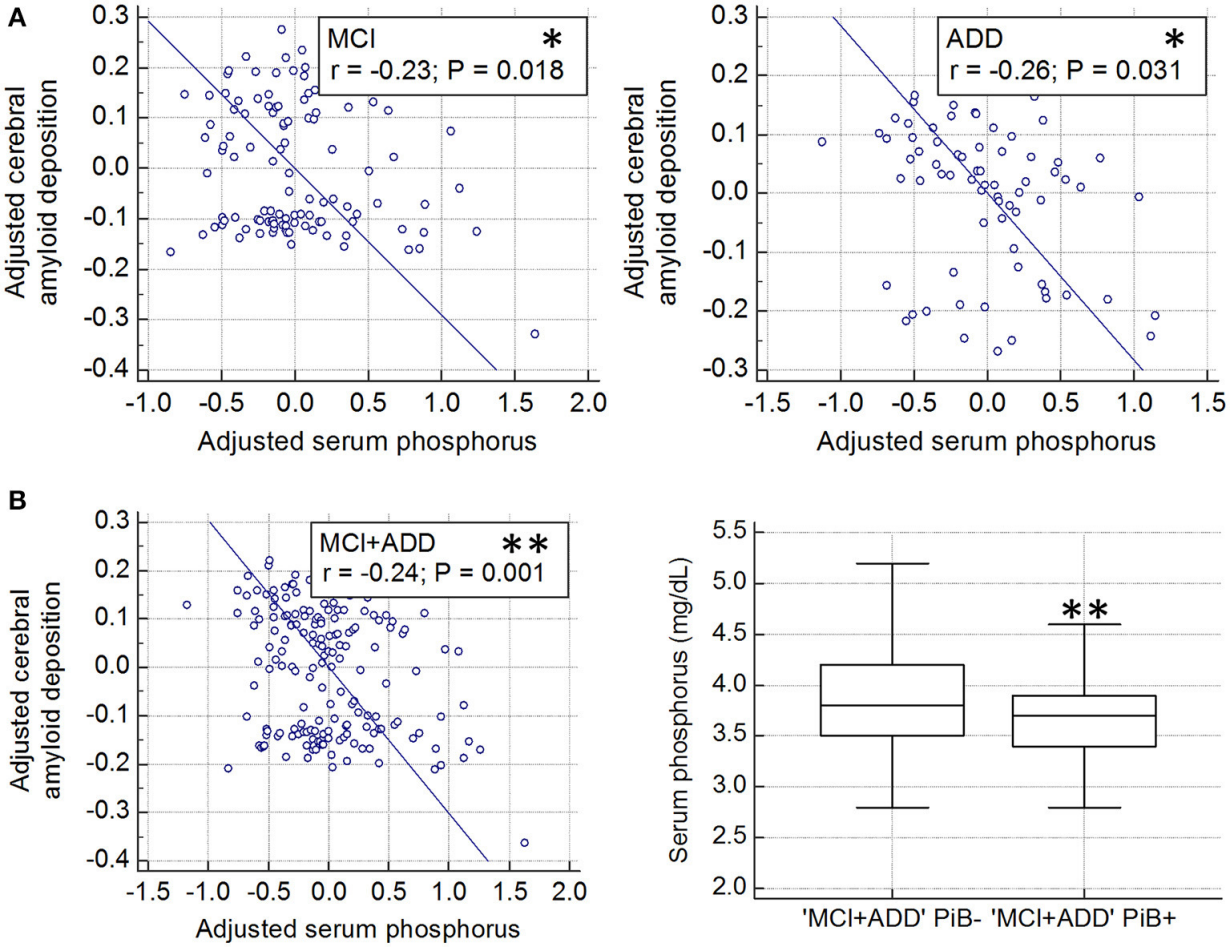
Association cerebral amyloid deposition with serum phosphorus levels in cognitively impaired subjects **(A)** Partial correlation plot shows the relationship between global cerebral amyloid deposition (SUVR) and serum phosphorus after controlling for age and sex, in both MCI and ADD (MCI, ^*^p < 0.05; ADD, ^*^p < 0.05). Global cerebral amyloid burden was natural log transformed to normalize variance. **(B)** Partial correlation plot in whole cognitively impaired subjects (MCI plus ADD, ^**^p < 0.01, left graph). Global cerebral amyloid burden was natural log transformed. Comparison of serum phosphorus levels between PiB+ and PiB− in whole cognitively impaired subjects (MCI plus ADD, ^**^p < 0.01; *P*–values were obtained by ANCOVA comparing adjusted mean after controlling for the effect of age and sex, right graph). PiB, Pittsburgh Compound B; MCI, mild cognitive impairment; ADD, Alzheimer's disease dementia; ±, PiB positivity; SUVR, standardized uptake value ratio.

The partial correlation analyses revealed a significant correlation between the serum level of phosphorus and the deposition of cerebral amyloid in both MCI (*r* = −0.23, *df* = 105, *p* = 0.018) and ADD (*r* = −0.26, *df* = 69, *p* = 0.031) groups (Figure 1A). A similar correlation was observed when the two groups were combined (*r* = −0.24, *df* = 178, *p* = 0.001; Figure 1B, left). In the combined (MC1 plus ADD) group, PiB+ subjects had a lower serum level of phosphorus as compared with PiB− subjects (*p* = 0.009; Figure 1B, right). To identify the relationship between the level of phosphorus and the deposition risk of cerebral amyloid, we conducted a relative risk (RR) analysis. We categorized subgroups into quartiles (quartile 1, ≤3.4 mg/dL; quartile 2, >3.4 and ≤3.7 mg/dL; quartile 3, >3.7 and ≤4.0 mg/dL; quartile 4, >4.0 mg/dL), as described in previous reports (Dhingra et al., 2007, 2010; Ye et al., 2016). The subjects with low levels of phosphorus (quartile 1, ≤3.4 mg/dL) showed higher risk of PiB positivity (RR = 1.50, *p* = 0.028; 95% CI, 1.04–2.14; Table 2). Chi-squared test for trend also showed that the proportion of PiB+:PiB− significantly decreased, as the quartile number increased (first quartile to fourth quartile) (*p* = 0.020, χ^2^ = 5.447). Furthermore, the multiple regression analyses were conducted in three models (set 1, MCI only; set 2, ADD only; set 3, both MCI and ADD; Table 3). Age, sex, and ApoE ε4 carrier status were included as covariates. Serum phosphorus showed a significant negative correlation with cerebral Aβ deposition in all three models (set 1, β = −0.193 and *p* = 0.037; set 2, β = −0.260 and *p* = 0.034; set 3, β = −0.226 and *p* = 0.003; Table 3). In addition, serum phosphorus level was correlated with cerebral Aβ deposition even after controlling for serum calcium levels and/or MMSE score with age, sex, and ApoE ε4 types (age, sex, calcium, and ApoE as covariates, β = −0.066 and *p* < 0.001; age, sex, MMSE score and ApoE as covariates, β = −0.066 and *p* < 0.001; age, sex, calcium, MMSE score and ApoE as covariates, β = −0.067 and *p* < 0.001; Table 4).

**Table 2:** Relationship between serum phosphorus and risk of cerebral amyloid deposition in cognitively impaired subjects.

**Serum phosphorus level**	**PiB status**		**RR (95% CI)**	***P*^‡^**	***P***
	**PiB−, *n* (%)**	**PiB+, *n* (%)**			
Quartile 1 (≤3.4 mg/dL)	16 (30.2)	37 (69.8)	1.50 (1.04 ~ 2.14)	0.0279^*^	^a^0.1020
Quartile 2 (>3.4 and ≤3.7 mg/dL)	14 (33.3)	28 (66.7)	1.43 (0.98 ~ 2.09)	0.0648	^b^0.0196^*^
Quartile 3 (>3.7 and ≤ 4.0 mg/dL)	16 (38.1)	26 (61.9)	1.33 (0.90 ~ 1.96)	0.1580	–
Quartile 4 (>4.0 mg/dL)	24 (53.3)	21 (46.7)	1	–	–

*PiB, Pittsburgh compound B; RR, relative risk; CI, confidence interval*.

P^‡^, P of RR;

a P by chi-square test χ^2^ = 6.207;

b P by chi-squared test for trend χ^2^ = 5.447;

* *p < 0.05*.

**Table 3:** Multiple regression analyses of phosphorus and global cerebral Aβ deposition.

**Covariates**	**β**	**SE**	***t***	***P*-value**	***F (df)***	***R^2^-adj***
**SET 1: MCI**						
^a^Dependent variable: global cerebral Aβ deposition				< 0.001^*^	6.71 (4, 103)	0.176
Age	−0.001	0.001	−0.867	0.388		
Sex	−0.030	0.092	0.323	0.747		
Phosphorus	−0.193	0.092	−2.111	0.037^*^		
ApoE ε4 type	0.393	0.086	4.589	< 0.001^*^		
**SET 2: ADD**						
^a^Dependent variable: global cerebral Aβ deposition				< 0.001^*^	5.52 (4, 68)	0.201
Age	−0.009	0.001	−1.392	0.168		
Sex	0.199	0.129	1.541	0.128		
Phosphorus	−0.260	0.120	−2.166	0.034^*^		
ApoE ε4 type	0.417	0.109	3.842	< 0.001^*^		
**SET 3: MCI** + **ADD**						
^a^Dependent variable: global cerebral Aβ deposition				< 0.001^*^	15.2 (4, 176)	0.240
Age	−0.008	0.005	−1.812	0.072		
Sex	0.100	0.077	1.298	0.196		
Phosphorus	−0.226	0.074	−3.030	0.003^*^		
ApoE ε4 type	0.457	0.067	6.813	< 0.001^*^		

a *Natural log-transformed to normalize variance*.

*Statistical significance*,

* *p < 0.05*.

*Aβ, beta-amyloid; MCI, mild cognitive impairment; ADD, Alzheimer's disease dementia; SE, standard error; β, regression coefficient; ApoE, Apolipoprotein E*.

**Table 4:** Multiple regression analyses of phosphorus, calcium, MMSE score, and ApoE ε4 genotype with global cerebral Aβ deposition in cognitively impaired subjects.

**Covariates**	**β**	**SE**	***t***	***P*-value**	***F (df)***	***R*^2^-adj**
^a^Dependent variable: global cerebral Aβ deposition				< 0.001^*^	14.0 (5, 175)	0.266
Age	−0.002	0.001	−1.848	0.066		
Sex	0.023	0.020	1.158	0.249		
Phosphorus	−0.066	0.019	−3.382	< 0.001^*^		
Calcium	0.028	0.024	1.173	0.242		
ApoE ε4 type	0.127	0.018	7.226	< 0.001^*^		
^a^Dependent variable: global cerebral Aβ deposition				< 0.001^*^	17.3 (5, 175)	0.312
Age	−0.003	0.001	−2.197	0.030^*^		
Sex	0.014	0.020	0.699	0.485		
Phosphorus	−0.066	0.019	−3.479	< 0.001^*^		
MMSE score	−0.007	0.002	−3.624	< 0.001^*^		
ApoE ε4 type	0.115	0.017	6.695	< 0.001^*^		
^a^Dependent variable: global cerebral Aβ deposition				< 0.001^*^	14.7 (6, 174)	0.313
Age	−0.003	0.001	−2.225	0.027^*^		
Sex	0.014	0.020	0.738	0.461		
Phosphorus	−0.067	0.019	−3.540	< 0.001^*^		
Calcium	0.027	0.023	1.181	0.239		
MMSE score	−0.007	0.002	−3.617	< 0.001^*^		
ApoE ε4 type	0.115	0.017	6.710	< 0.001^*^		

a *Natural log-transformed to normalize variance*.

*Statistical significance*,

* *p < 0.05*.

*Aβ, beta-amyloid; MCI, mild cognitive impairment; ADD, Alzheimer's disease dementia; SE, standard error; β, regression coefficient; ApoE, Apolipoprotein E*.

### Serum phosphorus increases discrimination power between PiB− vs. PiB+

**Figure 2:**
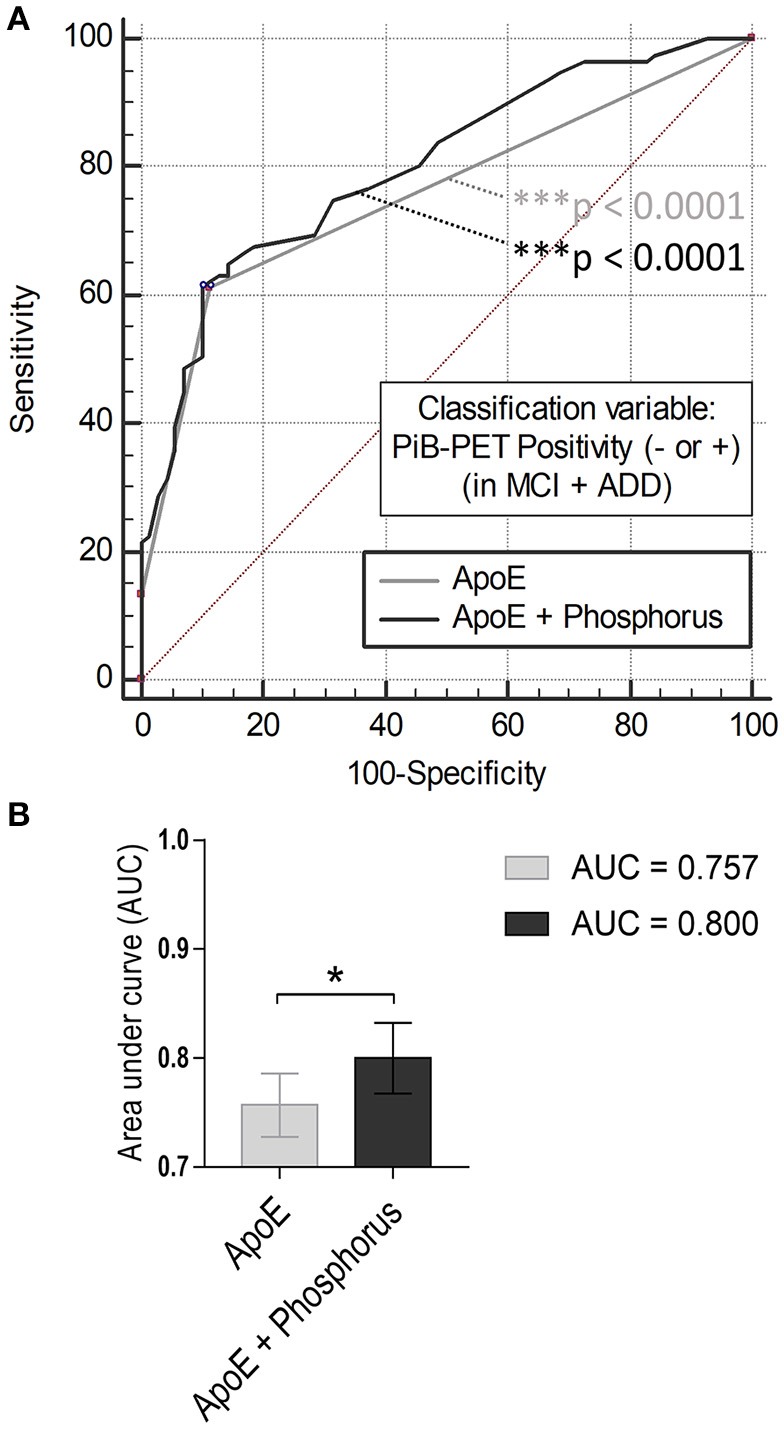
Comparison of ROC curves analysis **(A)** ROC curve models using independent variables (ApoE ε4 and/or serum phosphorus). Gray line (AUC: 0.757, ^***^p < 0.0001), ApoE alone; black line (AUC: 0.800, ^***^p < 0.0001). **(B)** Comparison of ROC curves analysis between the models (^*^p < 0.05, ApoE alone vs. ApoE with phosphorus).

We performed logistic regression and ROC curve analysis, using independent variables (ApoE ε4 carrier status and serum phosphorus, Figure 2). Each of the ROC curves had a significant *p*-value (*p* < 0.0001, Figure 2A). AUC value was significantly enhanced by combining the two variables (AUC, 0.757–0.800; Figure 2A, gray and black line; Figure 2B, *p* = 0.0343, comparison of ROC curves analysis). These results suggest that the serum level of phosphorus may be used as a blood-based biomarker for PiB positivity or AD in conjunction with ApoE ε4 genotype for cognitively impaired population.

### Other serum ion levels have no influence on either serum phosphorus or cerebral amyloid deposition

**Figure 3:**
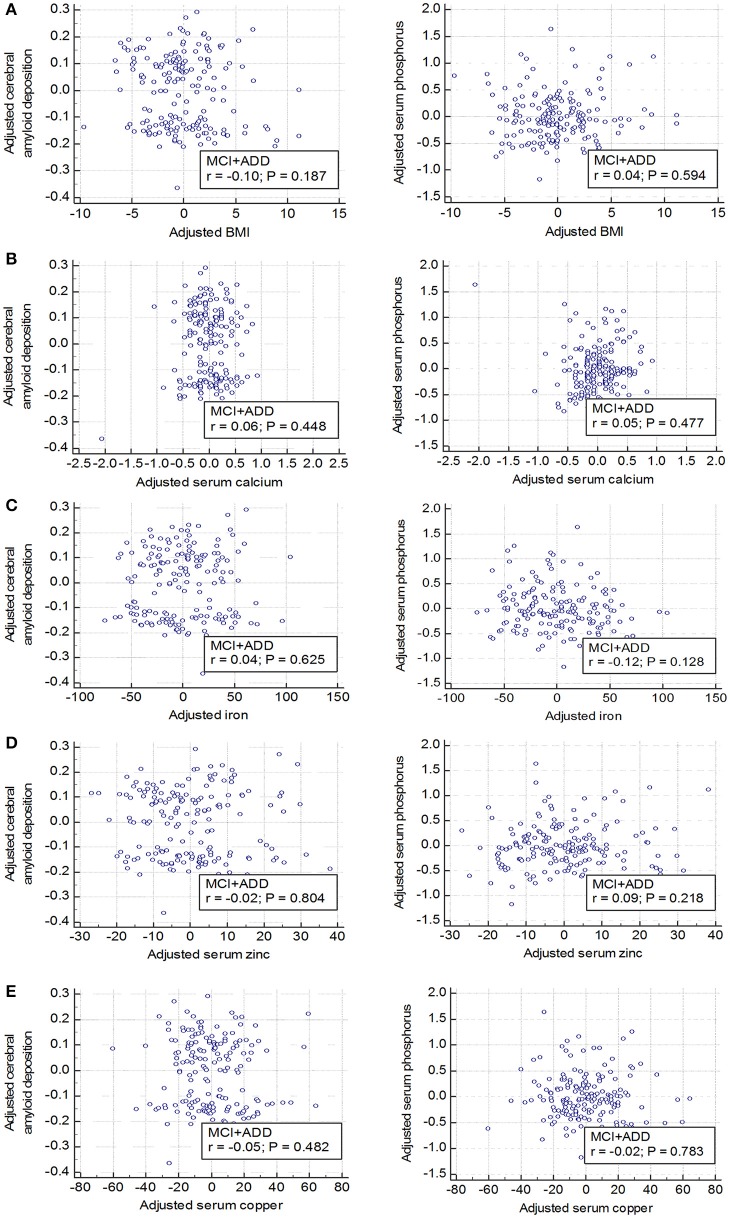
No relationship between elements and body mass index (BMI) and brain Aβ deposition or serum phosphorus **(A–E)** Elements (calcium, iron, zinc, and copper) and an indicator (BMI) which can reflect the health conditions are not associated with both cerebral amyloid deposition (left graphs) and serum phosphorus levels (right graphs). Age and sex were adjusted and global cerebral amyloid burden (SUVR) was natural log transformed to normalize variance. MCI, mild cognitive impairment; ADD, Alzheimer's disease dementia; SUVR, standardized uptake value ratio.

Partial correlation plots showed that the serum levels of other ions such as calcium, iron, zinc, and copper levels were not significantly correlated with either cerebral Aβ deposition (SUVR) (Figures 3B–E, left graphs; *p* > 0.05) or serum phosphorus levels (Figures 3B–E, right graphs; *p* > 0.05) after controlling for covariates, such as age and sex. Body mass index (BMI) showed no significant association with cerebral Aβ deposition either in cognitively impaired subjects (Figure 3A). In addition, multiple regression analyses showed that serum phosphorus was significantly associated with cerebral Aβ deposition even after controlling for covariates, including other ions and BMI (age, sex, BMI, calcium, iron, zinc, and copper as covariates, β = −0.072 and *p* < 0.01; Table 5). These results indicate that blood phosphorus level is associated with cerebral Aβ deposition, while other ion levels, including calcium, iron, zinc, and copper, are not correlated with brain Aβ deposition in cognitively impaired subjects.

**Table 5:** Multiple regression analyses of phosphorus, elements, and body mass index (BMI) with global cerebral Aβ deposition in cognitively impaired subjects.

**Covariates**	**β**	**SE**	***t***	***P*-value**	***F (df)***	***R*^2^-adj**
^a^Dependent variable: global cerebral Aβ deposition				< 0.001^*^	14.0 (8, 156)	0.037
Age	−0.002	0.001	−1.611	0.109		
Sex	0.033	0.026	1.283	0.202		
Phosphorus	−0.072	0.024	−3.020	0.003^*^		
BMI	−0.004	0.003	−1.158	0.249		
Calcium	0.034	0.028	1.199	0.233		
Iron	0.001	0.001	0.258	0.797		
Zinc	0.001	0.001	0.105	0.917		
Copper	0.001	0.001	−0.736	0.463		

a *Natural log-transformed to normalize variance*.

*Statistical significance*,

* *p < 0.05*.

*Aβ, beta-amyloid; SE, standard error; β, regression coefficient; BMI, body mass index*.

## Discussion

Phosphorus is an essential constituent of organic materials, including nucleic acids, organic phosphates, vertebrate teeth, and bones (Bansal, 1990). It plays an essential role in activating enzymes and generating energy as a component of molecules such as adenosine triphosphate (ATP). Diverse pathological states are related to the perturbation in metabolic homeostasis of phosphorus. Examples include not only peripheral muscle weakness and tremor but also neuropathological states such as metabolic encephalopathy, delirium, and seizures (Weisinger and Bellorin-Font, 1998; Subramanian and Khardori, 2000). It is therefore not surprising that phosphorus can affect human brain as well as regulation of peripheral metabolism. For instance, the interaction of phosphorus dendrimer with proteins related to neurodegenerative disease, such as prion, Aβ, and tau, has been shown to influence their aggregation process during pathogenesis (Andrasi et al., 1995; Wasiak et al., 2012). Based on these observations, it has been proposed that phosphorus affects the pathogenesis of neurodegenerative diseases (Andrasi et al., 1995; Wasiak et al., 2012).

The levels of phosphorus significantly decrease in both the brains (Andrási et al., 2005) and CSFs of AD patients (Subhash et al., 1991) as compared with controls. Thus, it is likely that the down-regulation of phosphorus in the brain is a consequence of the pathological processes rather than normal aging processes (Subhash et al., 1991). Intriguingly, recent studies proposed that increased levels of serum phosphorus contributed to the risk of ADD (Basheer et al., 2016; Li et al., 2017). In contradiction with these studies, however, we found that the subjects with serum phosphorus below 3.4 mg/dL (normal range 3.4–4.5 mg/dL) had a high risk of cerebral Aβ accumulation (Table 2). One explanation for this contradiction would be that the previous results were obtained from people at or under the age of 60. In these studies, no significant difference was observed among people over 60. In our study, the mean age of each cohort was over 70, suggesting that the difference may be attributed to the subjects' ages (Table 1). Indeed, Li et al. (2017) proposed that the role of serum phosphorus could be different in people with other dementias under 60 as compared with age-related dementia such as late-onset AD. Consistent with our speculations, when the data presented in the previous study (Li et al., 2017) are narrowed down to people at or over 60, the serum levels of phosphorus are reduced in incident dementia.

Amyloid aggregation and AD pathogenesis can be influenced by BMI as well as various ions such as calcium, iron, zinc, and copper are known to be relevant to amyloid aggregation (Miller et al., 2010; Hane et al., 2013; Han et al., 2016). The BMI value declines at the preclinical stage but not after the onset of AD (Gu et al., 2014). In this study, we found no significant correlation of cerebral Aβ deposition with serum levels of calcium, iron, and zinc (Figures 3B–E, left) or with BMIs (Figure 3A). The correlation between serum phosphorus level and cerebral amyloid deposition remained significant even after controlling for the effect of other ions and BMI in the multiple regression model (Table 5). Further studies are needed with larger sample sizes to examine the effects of more diverse elements and indices involved in phosphorus metabolism and nutritional states such as the diet pattern.

Cerebral Aβ deposition in AD is tightly associated with tauopathy characterized by hyperphosphorylation of tau in postmortem brains of AD (Morishima-Kawashima and Ihara, 2002; Bloom, 2014). Aβ induces tau phosphorylation via MAPK and GSK-3β, triggering microtubules destabilization, axonal transport impairment, and neuronal death (Busciglio et al., 1995; Greenberg and Kosik, 1995; Le et al., 1997; Takashima et al., 1998). Tau phosphorylation also contributes to Aβ-induced neurodegeneration, leading to the loss of septal cholinergic neurons (Zheng et al., 2002). Hyperphosphorylated tau needs tremendous amount of phosphorus (Iqbal et al., 2010), significantly depleting phosphorus from brains and blood (Butner and Kirschner, 1991; Khatoon et al., 1992). The disruption of the brain blood barrier may also contribute to decreases of phosphorus in both brains and blood during AD pathogenesis (Bell and Zlokovic, 2009; Zlokovic, 2011). However, knowledge of tau deposition and CSF tau concentrations would be needed to draw solid conclusions.

In this study, we used a natural log-transformed value of the global cerebral Aβ deposition (SUVR) as the dependent variable of multiple linear regression analyses to resolve non-normal distribution of global Aβ deposition value. However, the patterns of partial regression plots derived from multiple linear regression analyses seemed to roughly show bimodal distribution, even when the dependent variable, global Aβ deposition value, was natural log-transformed. This phenomenon might be related to characteristic bimodal distribution of cerebral Aβ deposition as reported in previous studies (Villain et al., 2012; Chételat et al., 2013; Villeneuve et al., 2015). However, given that our results from multiple logistic regression analyses were consistent with those from multiple linear regression analyses, our finding still supports the inverse relationship between serum phosphorus level and cerebral Aβ deposition. Further experiments are needed to elucidate the underlying mechanism. Following standard practice in PET image analysis, we used AAL to determine ROIs. Single-subject approaches such as AAL can be biased due to the idiosyncrasies of the atlas individual, therefore multi-subject approaches might be preferable (Rohlfing et al., 2004; Svarer et al., 2005; Heckemann et al., 2006). However, our analysis relied on four relatively coarse ROIs, therefore the benefit of using a more accurate segmentation method would have been small.

In conclusion, the combination of phosphorus with other biomarkers may help detect cerebral Aβ deposition more efficiently in cognitively impaired individuals with MCI and ADD. Our result suggests that the serum level of phosphorus may be used as an easily accessible blood biomarker for cerebral Aβ deposition in a cognitively impaired population.

## Ethics statements

The protocol was approved by the Institutional Review Board (IRB) of the Seoul National University Hospital and SMG-SNU Boramae Medical Center, Seoul, South Korea, and was carried out in accordance with the recommendations of the current version of the Declaration of Helsinki. All subjects or their legal representatives gave written informed consent in accordance with the Declaration of Helsinki.

## Author contributions

Study concept and design by J-CP, S-HH, DL, and IM-J. Acquisition of data by J-CP, S-HH, MB, DY, and JL. Analysis and interpretation of data by J-CP, S-HH, MB, DY, JL, DL, and IM-J. Critical revision of manuscript for intellectual content by KP, MB, DL, and IM-J. DL and IM-J supervised and coordinated all of the data in the study. All authors read and approved the final manuscript.

### Conflict of interest statement

The authors declare that the research was conducted in the absence of any commercial or financial relationships that could be construed as a potential conflict of interest.
