# Supplementary material for: Low Serum Phosphorus Correlates with Cerebral Aβ Deposition in Cognitively Impaired Subjects: Results from the KBASE Study
Source: Front Aging Neurosci. 2017 Nov 6;9:362. doi: 10.3389/fnagi.2017.00362 (PMC5681522; doi:10.3389/fnagi.2017.00362)
Supplement: Supplementary file 1 [file DataSheet1.DOCX]

**Appendix**

**KBASE Research Group**

Dong Young Lee, MD, PhD (Seoul National University, Principal Investigator); Min Soo Byun, MD, PhD (Seoul National University, Core PI Clinical & Executive); Dahyun Yi, PhD (Seoul National University, Core PI Neuropsychology); Yu Kyeong Kim, MD, PhD (SMG-SNU Boramae Medical Center, Core PI PET); Chul-Ho Sohn, MD, PhD (Seoul National University, Core PI MRI); Inhee Mook-Jung, PhD (Seoul National University, Core PI Biomarker); Murim Choi, PhD (Seoul National University, Core PI Genetics); Yu Jin Lee, MD, PhD (Seoul National University, Core PI Sleep), Seokyung Hahn, PhD (Seoul National University, Core PI Biostatistics); Hyun Jung Kim, MD (Changsan Convalescent Hospital, co-investigator); Mun Young Chang, MD (Chung-Ang University College of Medicine, co-investigator); Seung Hoon Lee, MD (Daerim St. Mary's Hospital, co-investigator); Jee Wook Kim, MD, PhD (Hallym University Dongtan Sacred Heart Hospital, co-investigator); Jong-Min Lee, PhD (Hanyang University, co-investigator); Dong Woo Lee, MD, PhD (Inje University Snaggye Paik Hospital, co-investigator); Bo Kyung Sohn, MD (Inje University Snaggye Paik Hospital, co-investigator); Seok Woo Moon, MD, PhD (Konkuk University Chungju Hospital, co-investigator); Man Ho Choi, PhD (Korea Institute of Science and Technology, co-investigator); Sang-Won Lee, PhD (Korea University, co-investigator); Hyewon Baek, MD (Kyunggi Provincial Hospital for the Elderly, co-investigator); Na Young Han, MD (National Research Center for Dementia, co-investigator); Jong-Won Kim, MD, PhD (Samsung Medical Center, co-investigator); Seung-Ho Ryu, MD, PhD (School of Medicine Konkuk University, co-investigator); Shin Gyeom Kim, MD, PhD (Soonchunhyang University Hospital Bucheon, co-investigator); Sun-Ho Han, PhD (Seoul National University, co-investigator); Jae Sung Lee, PhD (Seoul National University, co-investigator); Yun-Sang Lee, PhD (Seoul National University, co-investigator); Jong Inn Woo, MD, PhD (Seoul National University, co-investigator); Sang Eun Kim, MD, PhD (Seoul National University Bundang Hospital, co-investigator); Byung Chul Lee, PhD (Seoul National University Bundang Hospital, co-investigator); Gi Jeong Cheon, MD, PhD (Seoul National University Hospital, co-investigator); Koung Mi Kang, MD (Seoul National University Hospital, co-investigator); Jee-Eun Park, MD, PhD; (Seoul National University Hospital, co-investigator); Hyeong Gon Yu, MD, PhD (Seoul National University Hospital, co-investigator); Jun-Young Lee, MD, PhD (SMG-SNU Boramae Medical Center, co-investigator); Hyo Jung Choi, MD (SMG-SNU Boramae Medical Center, co-investigator); Young Min Choe, MD (University of Ulsan College of Medicine, Ulsan University Hospital, co-investigator); Woonhyung Ghim, MD (Seoul National University Hospital, research fellow); So Yeon Jeon, MD (Seoul National University Hospital, research fellow); Woo Jin Kim, MD, PhD (Seoul National University Hospital, research fellow); Kang Ko, MD (Seoul National University Hospital, research fellow); Jun Ho Lee, MD (Seoul National University Hospital, research fellow); Kyoungjin Chu (Seoul National University Hospital, psychologist); Hyunwoong Ko (Seoul National University Hospital, psychologist); Younghwa Lee (Seoul National University Hospital, psychologist); Donghwi Hwang (Seoul National University, image analyst); Seugn Kwan Kang (Seoul National University, image analyst); Seong A Shin (Seoul National University, image analyst); Jeong Yeon Hwang, MD (Seoul National University, data analyst); Jong-Chan Park (Seoul National University, data analyst); Jong-Ho Park (Samsung Medical Center, genetic data analyst); Jieun Seo (Seoul National University, genetic data analyst); Mi Ae Han (Seoul National University Hospital, research coordinator); Eun A Jo (Seoul National University Hospital, research coordinator); Gi Jung Jung (Seoul National University Hospital, research coordinator); Jin Hee Keum (Seoul National University Hospital, research coordinator); Mi Sun Kim (SMG-SNU Boramae Medical Center, research coordinator); Min Jeong Kim (Seoul National University Hospital, research coordinator); Han Na Lee (Seoul National University Hospital, research coordinator); Bo Eun Park (Seoul National University Hospital, research coordinator); Ji Sun Shin (Seoul National University Hospital, research coordinator); Yun Jung Hwang (Seoul National University Hospital, researcher); Joon Hyung Jung, MD (Seoul National University Hospital, researcher); Kiyoung Sung, MD (Seoul National University Hospital, researcher); Eun Hye Kim (Seoul National University, research assistant); Han Byul Choi (National Research Center for Dementia, administrative staff)

**KBASE Partner Organization**

Dongjak-gu Center for Dementia

Jongno-gu Center for Dementia

Ministry of Science, ICT and Future planning

National Research Center for Dementia

National Research Foundation of Korea

Seoul Metropolitan Center for Dementia

Seoul National University

Seoul National University Bundang Hospital

Seoul National University Hospital

SMG-SNU Boramae Medical Center

The Korean Association for Dementia
